# Electrochemical Analysis of the Corrosion Resistance of the Al-Alloy EN AW-5454-D and Its Welded Joints

**DOI:** 10.3390/ma19040750

**Published:** 2026-02-14

**Authors:** Matjaž Balant, Gyöngyi Vastag, Peter Majerič, Rebeka Rudolf

**Affiliations:** 1 Faculty of Mechanical Engineering, University of Maribor, Smetanova ulica 17, 2000 Maribor, Slovenia; matjaz.balant@student.um.si (M.B.); peter.majeric@um.si (P.M.); 2 Faculty of Sciences, University of Novi Sad, Trg D. Obradovica 3, 21000 Novi Sad, Serbia; djendji.vastag@dh.uns.ac.rs; 3 Zlatarna Celje d.o.o., Kersnikova ulica 19, 3000 Celje, Slovenia; 4 Jožef Stefan Institute, Jamova 39, 1000 Ljubljana, Slovenia

**Keywords:** Al-alloy, welding, electrochemical behaviour, characterisation

## Abstract

An electrochemical evaluation of the corrosion resistance of the Al-alloy EN AW-5454-D and its welded joints made by MIG (Metal Inert Gas) and by laser hybrid (LH) welding was performed in this study. All the tested samples had a thickness of 4 mm, whereby all the samples’ surfaces were cleaned with a plasma cleaning process before the electrochemical testing to reduce the impact of contamination. The electrochemical behaviour was investigated in a 3.5 wt.% NaCl electrolyte over exposure periods of 1 h, 7 days, and 30 days using electrochemical methods and surface examination. The results demonstrate that the welding processes (MIG and LH) caused microstructural heterogeneities that reduce the corrosion resistance of the weld. The MIG-welded specimen showed worse properties than the LH-welded specimen in the electrochemical tests, as it had a higher corrosion current density, lower polarisation resistance, and higher layer capacitance. Due to long-term exposure to the immersion solution, despite the reduced susceptibility to uniform corrosion, the Al-alloy samples and their welds remained susceptible to pitting corrosion.

## 1. Introduction

The properties of the EN AW-5454-D Al-alloy are well known from the scientific literature, such as very good corrosion resistance [1,2,3], good forming behaviour [4], favourable welding characteristics [5], good density-to-load capacity ratio [6,7], and excellent mechanical properties [3,8,9]. All these properties indicate that this Al-alloy is considered a strong candidate for automotive components [10] due to the reduction in weight and environmental footprint [11,12], and, consequently, lower energy consumption required to power vehicles [13]. Since this Al-alloy is corrosion-resistant and weldable, it is used for various chassis components that are joined together by welding.

Laser hybrid welding (LH) [14]: LH has many advantages [15] over conventional welding processes when choosing the optimal welding parameters since it offers higher processing speed and efficiency compared to conventional arc welding [16], provides lower heat input [17,18,19] into the welded joint, and, consequently, reduced microstructural modifications within the heat-affected zone (HAZ) [20,21,22], decreased energy input requirements, superior weld surface quality, and a reduction in welding-related defects [23,24]. Considering the size of the welding equipment, especially the LH welding head, which prevents access to more complex forms of welded parts, LH welding is best for welding long and straight welded joints. By contrast, the Metal Inert Gas (MIG) process offers distinct advantages, such as operational adaptability, high process efficiency, and favourable conditions for mechanisation and automation. The MIG welding process is used widely in automated welding [25]. Due to the relatively simple shape of the MIG torch, the accessibility to the welding site is much better than with LH welding and therefore allows the welding of more complex weld shapes. With the introduction of pulse MIG welding, the weld pool stirring is enhanced, and it allows a finer grain weld structure and better weld properties [25] in comparison to standard MIG welding. A key issue in MIG welding is the occurrence of porosity within the weld metal, which is greater than in LH welding [26,27]. A systematic review conducted by Saifudin et al. [28] argues that Laser–MIG hybrid welding can reduce the porosity in Al welds by appropriate adjustments to the heat input and speed. This review provides literature support for our claim that lower porosity in LH contributes to reduced ionic permeability and more stable barrier layers, consistent with our EIS-based corrosion ranking, LH > MIG.

The Al-alloy designated EN AW-5454-D is included in the 5000 series Al-Mg alloys of work-hardening Al-alloys [29,30]. With thinner sheet metal, the material is more hardened, and therefore has better mechanical properties, which was confirmed in a previous study of the same Al-alloy [27,31].

Thermal input during welding causes microstructural modifications within the heat-affected zone (HAZ), which, in turn, influence the material’s corrosion resistance [32,33,34]. The literature reports numerous studies examining factors that influence the corrosion resistance of Al-alloys. Bunaziv et al. [35] made a comprehensive review of laser beam and laser–arc hybrid welding of Al-alloys, detailing key challenges such as porosity, keyhole stability, and weld heterogeneity, and how hybridisation can mitigate the typical arc welding defects. This review contextualises our finding that LH > MIG in corrosion performance by linking hybrid welding to lower porosity/heterogeneity, which we then quantified mechanistically via EIS (C_2_, R_2_) on EN AW 5454 D. In his study, Sinan [1] investigated how rolling-based plastic deformation affects corrosion resistance, reporting an improvement in the corrosion resistance of the deformed Al-alloy. The effect of the degree of deformation on corrosion was investigated using a potentiodynamic test, although such a change was also recorded by corrosion testing. Fraker et al. [2] assessed the corrosion performance of several Al-alloys at elevated temperatures in salt water, and found that the AA5454 Al-alloy was the best among the Al-alloys studied. Yang et al. [36] investigated how changes in welding speed affect the aluminium–lithium alloy (Al-Li) 2195 in an LH process, finding that higher welding speeds led to lower corrosion resistance. In their study, Chandra et al. [37] investigated the impact of friction stir welding (FSW) on the corrosion behaviour of welded EN AW-7075 Al-alloy joints. Frankel & Xia [38] observed how friction stir-welded and arc-welded joints on an AA5454 Al-alloy exhibit different localised corrosion/SCC susceptibilities; FSW generally outperforms arc welding in pitting resistance, with weld process-dependent microstructures driving the differences. Their study underpins our observed hierarchy (BM > LH > MIG) by demonstrating that an arc-welded Al-alloy can be more prone to localised attack, which our EIS (higher C_2_, lower R_2_ in MIG) captures after 30 days. Guzmán et al. [39] conducted a comprehensive investigation of the electrochemical corrosion properties and mechanical performance of 6061 aluminium welds in two different heat treatments. The welds were made by MIG arc welding in pulsed mode. Microstructural and phase composition changes led to the formation of a galvanic couple prone to electrochemical corrosion. This effect was more pronounced in the heat-affected zone, whereas the remaining weld regions exhibited predominantly uniform and localised corrosion, as confirmed by electrochemical impedance spectroscopy. The thermal input applied during welding, especially in the HAZ, affects the corrosion resistance negatively. Shah and Badheka [40] evaluated the electrochemical behaviour of the AA 7075-T651 Al-alloy under different process parameters on corrosion performance. The corrosion performance was investigated in compliance with ASTM G-34. The outcomes demonstrated that the heat-affected zone (HAZ) represented the region most prone to corrosion attack. The experimental observations established further that the corrosion resistance of the welded specimens was strongly dependent on the magnitude of the heat input during welding. Welds produced using a lower tool rotational speed, higher welding speed, and a smaller tool diameter displayed superior corrosion resistance. These findings indicate collectively that heat input is the principal parameter governing the corrosion resistance of welded joints. Notably, the lowest corrosion resistance was recorded consistently in the HAZ. Zhou et al. [41] made a comparative study of 5xxx Al–Mg alloys in 3.5 wt.% NaCl, demonstrating passivation with long-term pitting initiation at the intermetallics, and showing how the alloying content shifts the electrochemical metrics (e.g., polarisation resistance, pitting potential). This study supports our pitting-focused interpretation and our use of EIS to separate the outer/inner layer behaviour, aligning with our observation that the microstructure controls the ionic ingress and barrier layer stability in chloride media.

Welded structures from the EN AW-5454-D Al-alloy are used widely in automotive applications, where the winter service exposes underbody components to chloride-bearing aqueous films formed from de-icing salts; warming cycles in garages accelerate corrosive attack further. To reflect these realistic conditions, we subjected both the base Al-alloy and welded joints to controlled chloride exposure and evaluated their corrosion performance in a unified, side-by-side framework.

In this study, we provide a direct, side-by-side corrosion comparison of MIG and LH welds on the EN AW-5454-D Al-alloy in 3.5 wt.% NaCl at three exposure times (1 h/7 d/30 d) [35]. We couple potentiodynamic polarisation with EIS, and derive the outer/inner layer parameters (e.g., C_2_, R_2_) to show how the choice of welding process governs the ionic ingress through the porous outer products and the stability of the inner barrier layer, and thus the propensity for pitting [41]. Specifically, after 30 days, the MIG-welded Al-alloy exhibited a distinctly higher inner-layer capacitance (C_2_) than the LH-welded specimen, which we related to higher permeability and local activation [38]. This fills a gap in prior hybrid-welding work that focused mainly on porosity/mechanics and on other Al series, by adding a quantitative EIS–microstructure link for an EN AW-5454-D Al-alloy [28].

## 2. Materials and Methods

### 2.1. Base Material

The Al-alloy was obtained from Speira GmbH (Grevenbroich, Germany) as cold-rolled sheet strips with a thickness of 4 mm, delivered in the O-temper (soft, annealed) [4]. The longitudinally rolled plates were cut to the required dimensions of 50 mm × 500 mm.

Table 1 shows the nominal chemical composition of the supplied EN AW-5454 alloy.

### 2.2. Filler Material

A massive welding wire, Safra Al Mg2.7Mn (S Al5554—EN ISO 18273 [43]), with a wire diameter of Ø 1.2 mm, supplied by the manufacturer Safra Spa (Travagliato, Italy), was used for welding.

Table 2 shows the chemical composition of the welding wire supplied for the experimental work.

### 2.3. Welding

**Figure 1:**
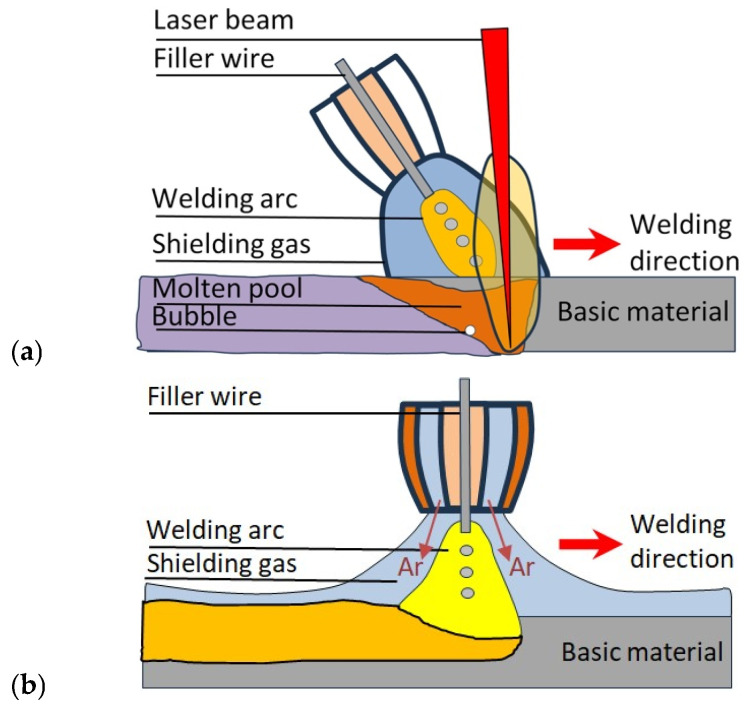
Schematic representation of the elements during (**a**) LH and (**b**) MIG welding.

**Figure 2:**
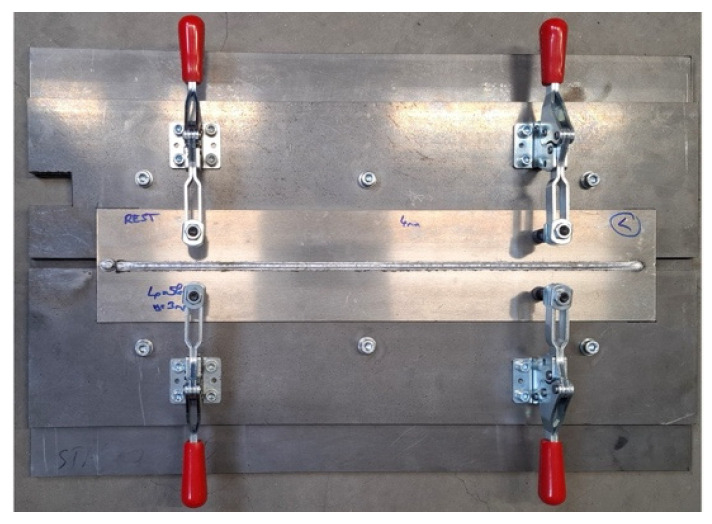
Weldments are clamped in the welding fixture.

The Al-sheet samples were welded using a robotic system in a designated welding cell to ensure operational safety. The individual elements during the LH and MIG welding are shown in Figure 1. The use of robotic welding required accurate positioning and clamping of the weldments in the fixture, as illustrated in Figure 2.

#### 2.3.1. Performing LH Welding

A laser welding source IPG YLS-10000-S2T with Laser process (continuous, pulsing, Fibre, solid): CW (Continuous Welding) Mode (IPG Laser GmbH, Allershausen, Germany), a welding power source for the synergic MIG welding process: PMC 4181 AC Universal: Fronius iWave500 (Fronius, Wels, Austria), together with a Fronius LH welding head (Fronius, Wels, Austria), were employed for the LH. The LH welding parameters for the butt weld are provided in Table 3.

#### 2.3.2. Performing MIG Welding

A welding source, Fronius TPS 400 i (Fronius, Wels, Austria) with Synergic Line: PMC 4515 DC Universal, was employed for the MIG process. The MIG welding parameters for the butt weld are provided in Table 3.

### 2.4. Sample Preparation

Rectangular samples were cut roughly by wire erosion from the base material (BM) and the LH- and MIG-welded samples. The shape of the samples of the base material was processed mechanically from a rectangle—the contour of the base material sample imitated the contour (outline) of the welded sample, so that the surface sizes of all the samples were as equal as possible. The contour in the base material was then created with an electric straight sander, and later with a hand file according to the outlined contour.

Holes with a diameter of 1.0 mm were drilled into the samples mechanically, which were used to hang them on a wire in the test medium.

### 2.5. Characterisation

Microstructural observations and microchemical analyses of the samples were performed using a Scanning Electron Microscope (SEM), model Sirion 400 NC (FEI, Hillsboro, OR, USA), equipped with an Energy-Dispersive X-ray (EDX) spectroscope, INCA 350 (Oxford Instruments, Tubney Woods, Abingdon, Oxfordshire, UK). SEM imaging was performed in both secondary electron (SE) and backscattered electron (BSE) modes at magnifications from 500× to 2500×. An acceleration voltage of 20 kV and an electron beam spot size of 3.0–4.0 were employed to optimise the resolution and contrast. An EDX analysis was carried out over an energy range of 0–20 keV to determine the elemental composition of selected areas. The results were normalised to provide quantitative elemental data.

The BM and LH- and MIG-welded samples were analysed after mechanical processing, chemical and mechanical cleaning, as well as after cleaning with air plasma. Additional SEM investigations were then conducted on the samples after the electrochemical testing. ImageJ software, version 1.54p, was used for image processing and evaluating the amount of surface area of the pitting corrosion.

### 2.6. Sample Surface Cleaning

**Figure 3:**
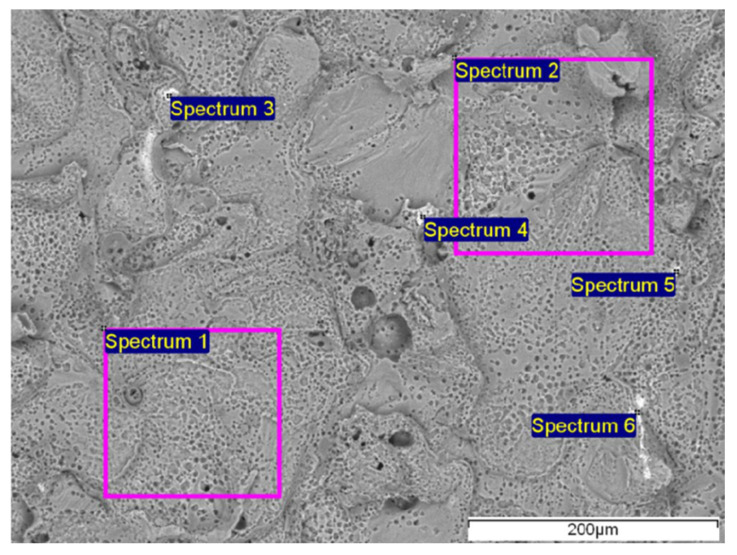
SEM analysis of the LH weld cross-section with marked areas of the EDX analysis, for spectrum 1–6 in Table 4.

After mechanical processing, the samples were examined by SEM/EDX, which revealed the remains of a cutting tool on the surface (the sample was cut with a hand file-cutting tip made of austenitic steel). Fe was detected on the surface, visible as white particles in Figure 3. Different amounts of Fe were present at the analysed sites. Fe, and in some places Cr, and Mo, were detected (Table 4).

Different cleaning approaches were used to remove these residual elements. The sample surfaces were examined with SEM following the various cleaning approach steps, to confirm that the residual elements had been removed from the sample surfaces.

#### 2.6.1. Chemical Cleaning

The specimens were etched in a 2 vol.% Nital solution (2 vol.% HNO_3_: 98 vol.% ethanol) etchant for 1 min, and then rinsed with water and dried.

An EDX examination of the samples after the cleaning attempt detected that different amounts of Fe were still present at the analysed sites.

#### 2.6.2. Mechanical Cleaning

Manual polishing of the samples was carried out with Al_2_O_3_. The samples were sanded with a 4000 base—each sample for about 10 min. Following the sanding step, the specimens were subjected to ultrasonic cleaning for 5 min. Subsequent polishing was then conducted using 0.05 µm MasterMet colloidal silica paste, approximately 5 min/side/sample.

After the 2nd cleaning attempt with the Nital solution, the samples were examined by EDX, which showed no more Fe residues on the surface.

#### 2.6.3. Cleaning with Air Plasma

Surface cleaning with air plasma was performed to remove organic contamination from the specimen surfaces, which could otherwise affect the corrosion resistance measurements. A Plasmatreat FG5001 plasma generator, RD1004 nozzle, without rotation, and a table with automated scrolling (Plasmatreat GmbH, Queller Straße 76-80, 33803 Steinhagen, Germany) were used for the surface conditioning.

The compressed-air plasma jet acts primarily as a cleaning and activation tool: the reactive oxygen-containing species generated in the discharge remove the adsorbed hydrocarbons and increase the density of the surface hydroxyl groups. This results in a higher-energy, more wettable surface, while the thickness of the native Al_2_O_3_/AlOOH passive film remains essentially unaffected under the mild, air-only settings used in this study. The enhanced hydrophilicity exhibits partial relaxation over time; therefore, all the electrochemical tests were initiated immediately after the plasma treatment to maintain a consistent starting condition [44,45,46].

The plasma activation was employed solely to establish a reproducible and contamination-free surface before immersion in the chloride electrolyte. Under the specific operational parameters listed in Table 5, the jet desorbs organic species without modifying or thinning the native oxide film, thereby improving the surface uniformity across the BM and welded specimens. The removal of handling-related residues improves the wettability and reduces the sample-to-sample variations [44,45].

To prevent the surface ageing effects associated with delayed measurement, all the electrochemical experiments were performed immediately after cleaning. This standardisation step avoids uncontrolled contaminants being superimposed on the chloride environment, and ensures that the observed differences between the MIG- and LH-welded joints originate from weld-related microstructural features rather than from inconsistent surface conditions [44,45].

**Figure 4:**
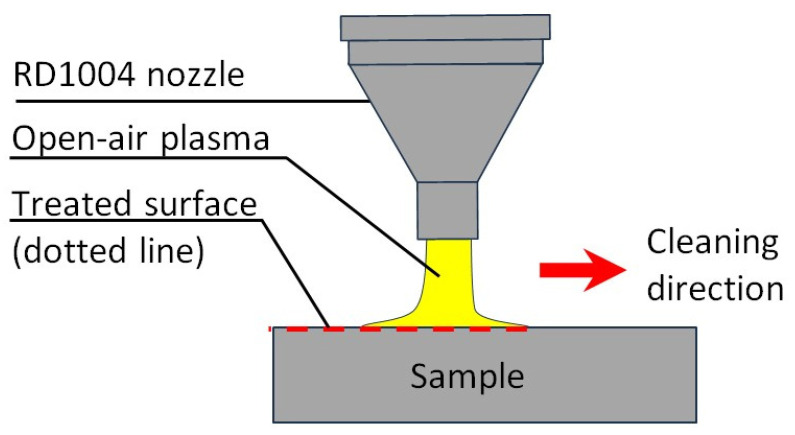
Surface preparation with air plasma.

Table 5 provides the technological specifications for the surface preparation procedure. The sample surface preparation with air plasma is presented in Figure 4.

### 2.7. Sample Surface Area Size Determination

**Figure 5:**
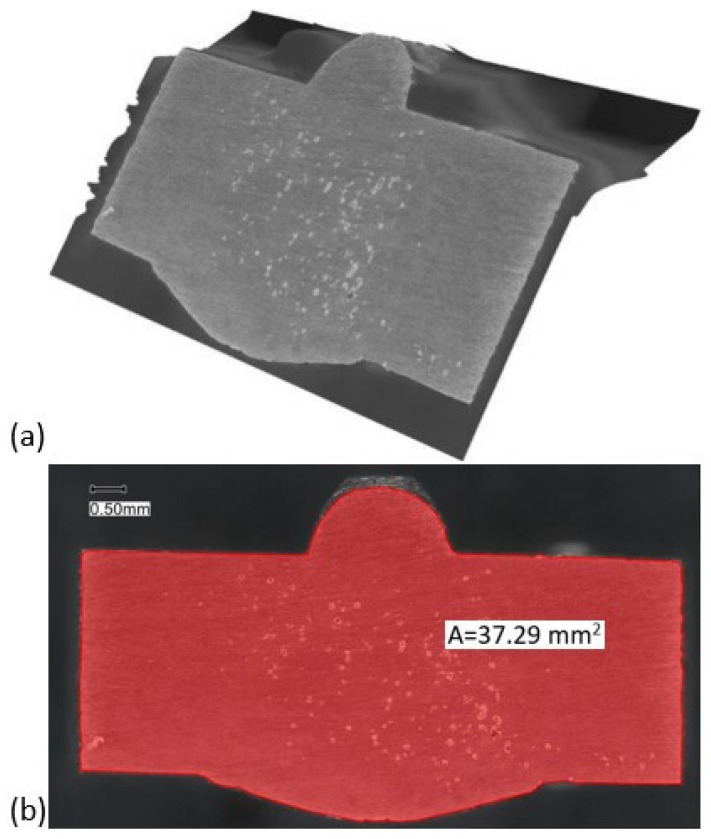
Surface area size determination: (**a**) 3D visualisation of the sample, (**b**) 2D visualisation of the sample.

To calculate the polarisation and electric potential, it was necessary to determine all the surfaces of the samples accurately; therefore, the surfaces were captured in 3D using a Keyence VHX-7000 digital microscope (Keyence International, Mechelen, Belgium) with 100× magnification. With the included software, a 2D image was made from the 3D image, as shown in Figure 5. A Poligon function was used to draw a closed curve (points at 0.01 or 0.1 mm) to 0.01 mm precision, and the Area function was used to determine the area (interpolation). An approximation was made on the other side of the sample (×2 of the measured area).

### 2.8. Electrochemical Testing Procedures

The electrochemical behaviour of three Al-alloys, the BM, LH-welded, and MIG-welded samples, was investigated in a 3.5 wt.% NaCl solution over immersion periods of 1 h, 7 days, and 30 days. The chemical compositions of the tested Al-alloys are listed in Table 6. The NaCl solution was prepared using analytical grade NaCl and distilled water. The electrochemical measurements were performed using a conventional three-electrode cell in a 400 mL open-air beaker. The constant composition of the NaCl solution during the measured period was maintained by monitoring the conductivity of the solution constantly. The working electrode was the tested Al-alloy sample, the counter electrode was a platinum mesh, and the reference electrode was a saturated calomel electrode (SCE). All the potentials are reported versus the SCE. The electrochemical measurements were carried out using a VoltaLab PGZ-301 potentiostat (Radiometer Analytical (A Hach Company Brand), Villeurbanne Cedex, France). Before each test, the open circuit potential (OCP) was monitored for 60 min to ensure that a stable steady state condition was reached. Following the OCP stabilisation, electrochemical impedance spectroscopy (EIS) was conducted at the OCP over a frequency range of 0.1 Hz to 30 kHz with a sinusoidal perturbation amplitude of 10 mV. The EIS data were fitted using the appropriate equivalent circuit models. In the potentiodynamic polarisation tests, the potential was scanned from −1100 mV to 500 mV at a sweep rate of 1 mV/s. The corrosion potential (E_corr_), corrosion current density (j_corr_), and polarisation resistance (R_p_) were determined from the polarisation curves using Tafel extrapolation, using VoltaMaster 4 software. All the measurements were performed in triplicate.

## 3. Results and Discussion

### 3.1. SEM Imaging and EDX Microchemical Analysis

After the mechanical processing of the samples, residual elements from the cutting tool were detected on the surface, which may interfere with further investigations and the electrochemical testing results.

**Figure 6:**
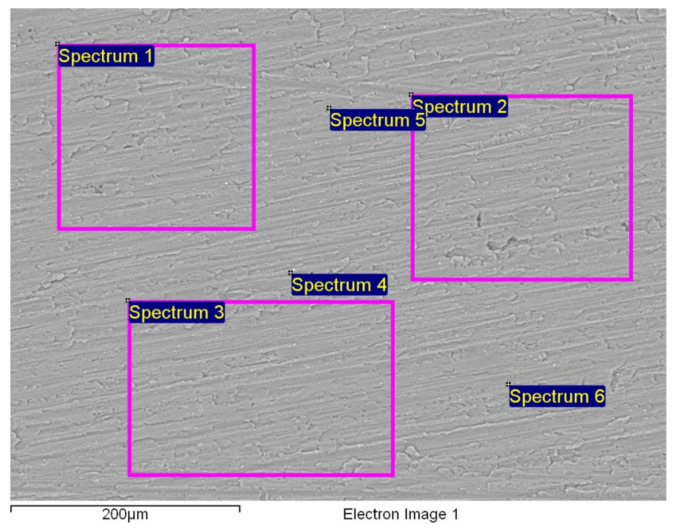
SEM analysis of the BM samples with marked areas of the EDX analysis, for spectrum 1–6 in Table 6.

The SEM/EDX investigations of the BM samples after successful cleaning of the cutting residuals are shown in Figure 6 and Table 6. The obtained EDX results confirm that the surface chemistry is consistent with the expected composition of the EN AW 5454 D Al-alloy. The detected oxygen originated from the natural Al_2_O_3_ passive film, which forms spontaneously upon the exposure of Al to air, and is observed commonly in EDX due to its limited analysis depth, rather than from surface contamination.

Importantly, no Fe, W, Mo, Cr, or V were detected after cleaning, demonstrating that the procedure removed the tool-related contaminants effectively. The concentrations of the key alloying elements (Mg, Mn, and Si) aligned well with the nominal compositional specification for EN AW 5454 D. Furthermore, the low Standard Deviation among the individual spectra indicates minimal spatial variation and confirms that the surface is cleaned uniformly and chemically homogeneous.

### 3.2. Electrochemical Measurements

#### 3.2.1. Open-Circuit Potential Measurement

**Figure 7:**
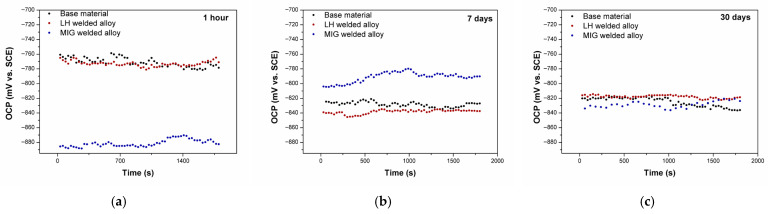
OCP values for the tested Al-alloys following immersion in a 3.5 wt.% NaCl solution: (**a**) 1 h, (**b**) 7 days, and (**c**) 30 days.

Figure 7 presents the open circuit potential (OCP) of the tested Al-alloys as a function of the immersion time in a 3.5 wt.% NaCl solution. During the first hour, the Al-alloys exhibited stable OCP values with fluctuations within ±20 mV, indicating rapid surface layer formation. The BM displayed the noblest potential (around −750 mV), while the MIG-welded Al-alloy showed the most active potential (around −806 mV).

With extended immersion (7 and 30 days), a marked cathodic shift of OCP was observed for most Al-alloys. This kind of behaviour suggests that gradual electrochemical activation of the Al-alloy surface is a function of time. The exception was the MIG-welded sample after 7 days, which shifted slightly anodically, possibly due to temporary re-passivation. After 30 days, the OCP values converged within a narrow range (−815 to −795 mV), implying similar electrochemical surface states across all the Al-alloys.

#### 3.2.2. Potentiodynamic Measurements

**Figure 8:**
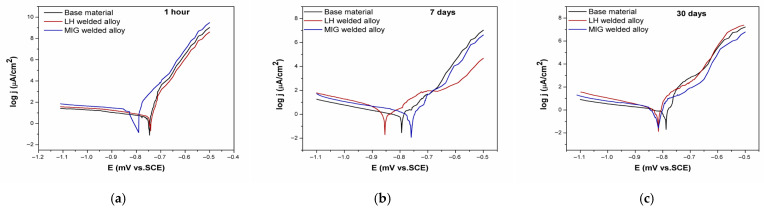
Polarisation curves of the tested Al-alloys for different immersion times in a 3.5 wt.% NaCl solution: (**a**) 1 h, (**b**) 7 days, and (**c**) 30 days.

The potentiodynamic polarisation curves of the tested Al-alloys immersed at different times in the 3.5 wt.% NaCl solution are shown in Figure 8. The corrosion parameters derived from potentiodynamic polarisation measurements were calculated by the Tafel equation. The obtained average values for corrosion potential (E_corr_), corrosion current density (j_corr_), and polarisation resistance (R_p_) are summarised in Table 7 for immersion times of 1 h, 7 days, and 30 days in a 3.5 wt.% NaCl solution.

At the onset of the immersion (1 h), the BM and LH-welded Al-alloy exhibited similar corrosion potentials (approximately −750 mV). In contrast, the MIG-welded Al-alloy showed a significantly more negative corrosion potential (−806 mV), indicating its lower initial corrosion resistance. This was further confirmed by its considerably higher corrosion current density (18.15 µA/cm^2^) and lower polarisation resistance (1.60 kΩ cm^2^) compared to the base Al-alloy (3.80 µA/cm^2^; 4.55 kΩ cm^2^). The inferior initial performance of the welded Al-alloys, particularly MIG, is attributed to the microstructural heterogeneities and porosity introduced during the welding process, which increased the electrochemically active surface area and facilitated charge transfer reactions.

With prolonged immersion (7 days), the corrosion potential of the BM and LH-welded Al-alloys shifted towards more negative values, suggesting gradual activation of the surface. At the same time, in the case of the LH-welded Al-alloy, a much larger shift was recorded than in the case of the BM.

Following 30 days of immersion, the corrosion potential (E_corr_) of the BM remained stable relative to its 7-day measurement. In contrast, the E_corr_ of the LH-welded Al-alloy shifted to more positive values during this period. This trend implies that the initially active surface observed at 7 days was subsequently passivated, likely due to the formation of corrosion products that reduced the electrochemical activity.

Conversely, the MIG-welded Al-alloy exhibited a different behaviour. Its corrosion potential shifted positively between 1 h and 7 days (from −806 mV to −761 mV), indicating an initial stabilisation or onset of passivation on the previously active surface. However, this positive shift did not persist. By day 30, the potential trend had reversed, suggesting a degradation of the protective effect that had begun to form earlier.

However, after 30 days, the corrosion potential of all the tested Al-alloys converged within a narrow range (approximately −800 mV), implying that the surface coverage with corrosion products became similar over time, regardless of the initial BM or weld microstructure.

The polarisation resistance (R_p_) increased substantially for all the Al-alloys after 7 days, indicating improved resistance to uniform corrosion. This trend correlates with the marked decrease in corrosion current density over the same period, reflecting the formation of a more protective surface layer. A similar trend was registered after 30 days.

Throughout the test intervals, the base Al-alloy maintained the best corrosion properties in terms of uniform corrosion. Although the LH-welded Al-alloy showed slightly better performance than the MIG-welded Al-alloy, after 30 days, the difference between the two welded variants was much smaller than at the beginning of the experiment. Notably, after 30 days, the MIG-welded Al-alloy exhibited a lower R_p_ value, which may be due to the development of a porous corrosion product layer within the weld structure.

Comparing the Rp value obtained from the polarisation measurements (Table 7) with the total resistance calculated from the EIS measurements (Table 8), it is noticeable that the obtained values are in good agreement. Namely, the established hierarchy of performance persisted with both methods of determining Rp.

The slight increase in j_corr_ observed for all the Al-alloys after 30 days may signal the gradual degradation of the protective surface layer, potentially heralding the onset of localised corrosion processes.

A decrease in the current density over time typically signals a reduction in uniform corrosion, but, in electrolytes containing aggressive ions like chloride (Cl^−^), this trend alone does not rule out the initiation or intensification of localised pitting corrosion [47,48].

For assessing the pitting formation, the key electrochemical parameters are most critical: the pitting potential (E_pit_) and the re-passivation or protective potential (Eᵣₑₚ) [47,49]. The relationship between these two values is especially significant. A sharp, premature rise in anodic current density before reaching oxygen evolution potentials often suggests instability in the passive oxide layer and the likely onset of pitting (E_pit_). The protective potential (E_rep_) is identified during a reverse potential scan as the point where the current hysteresis loop closes, i.e., where the return scan intersects the forward scan [47]. The measured re-passivation potential (E_rep_) defines a critical threshold below which new pits should not initiate.

To assess whether the formed layer on the Al-alloy surface after a different exposure period in a 3.5 wt.% NaCl solution could protect the tested Al-alloys against pitting corrosion, a potentiodynamic polarisation test was performed, which included a reverse potential scan.

**Figure 9:**
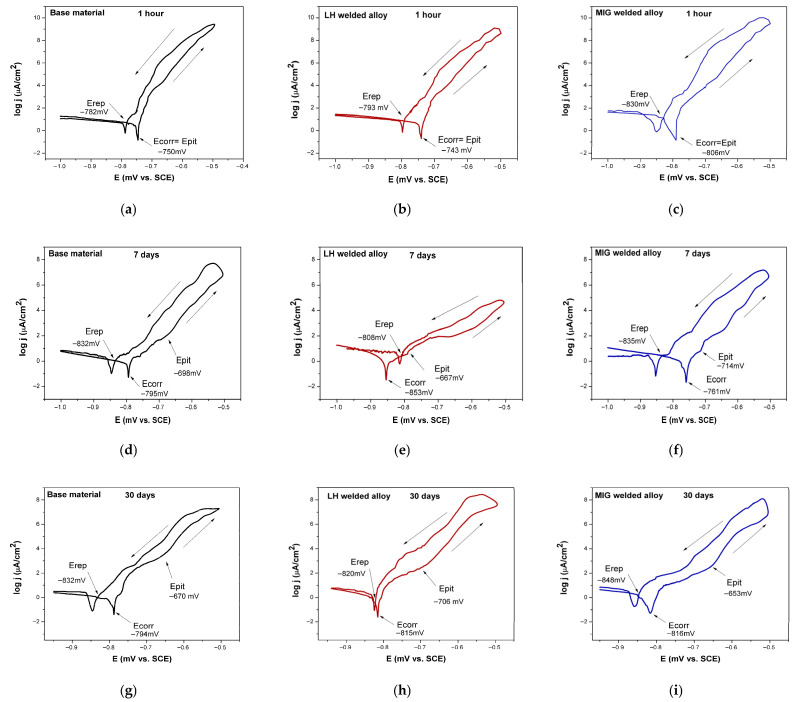
Polarisation curve with reverse scan of the tested Al-alloys after an immersion time of 1 h (**a**–**c**), 7 days (**d**–**f**), and 30 days (**g**–**i**) in 3.5 wt.% NaCl. The arrow indicates the direction of polarization.

Figure 9a–i present the potentiodynamic polarisation curves, including reverse scans, for the BM, LH-welded, and MIG-welded Al-alloys after 1 h, 7 days, and 30 days of immersion in the 3.5 wt.% NaCl solution. The parameters of the reversed scan polarisation measurements (E_corr_, E_pit_, E_rep_) of the tested Al-alloys are summarised in Table 8 for the immersion times of 1 h, 7 days, and 30 days in a 3.5 wt.% NaCl solution.

It is noticeable in Figure 9a–i that in all the immersion times in the 3.5 wt.% NaCl solution, all the tested Al-alloys exhibited a higher current density during the reverse scan compared to the forward scan. This resulted in a positive hysteresis loop, a characteristic electrochemical indicator that pitting corrosion has initiated under these conditions. The difference between the E_pit_ and E_rep_, along with the area of the hysteresis loop, is an indicator of pitting corrosion possibility. The larger E_pit_ and E_rep_ difference and the larger positive loop area correspond to lower pitting corrosion resistance [38].

At the initial 1 h immersion (Figure 9a–c), the corrosion potential (E_corr_) and pitting potential (E_pit_) were virtually identical for all the Al-alloys. Due to their high reactivity in air, metals like aluminium develop a surface oxide layer spontaneously, even prior to electrochemical polarisation. As a direct consequence, an increase in current density can be observed, beginning at, or very near, the corrosion potential. This behaviour results in the measured corrosion potential (E_corr_) and pitting potential (Eₚᵢₜ) being identical or extremely close in value at the beginning of the immersion [49,50,51].

At this early stage, E_rep_ was positioned more negatively than E_corr_ for all the Al-alloys (Figure 9a–c), indicating that any initiated pits were unlikely to re-passivate and would continue to propagate [38].

After 7 days (Figure 9d–f), a separation between E_corr_ and E_pit_ emerged, signalling the development of a more stable protective layer. The pitting potentials shifted to more noble values, with the LH-welded Al-alloy exhibiting the most positive E_pit_ and the MIG-welded Al-alloy the most negative. A critical behavioural divergence was observed in the LH Al-alloy, where E_rep_ shifted to a value between E_corr_ and E_pit_ (Figure 9e). This positioning creates a stable potential window (between E_corr_ and E_rep_) where the passive film is maintained, preventing both the initiation and growth of new pits [38].

This performance hierarchy was maintained after 30 days of immersion (Figure 9g–i). For the BM and MIG-welded Al-alloy, E_pit_ continued to drift positively, while the E_rep_ of the MIG-welded Al-alloy shifted negatively over time. The resulting expansion of the E_corr_ − E_rep_ difference signifies a progressive decrease in pitting corrosion resistance with prolonged exposure [38]. In contrast, the LH-welded Al-alloy exhibited a distinct behaviour (Figure 9h). After 30 days, its E_rep_ (−820 mV) remained nearly identical to its E_corr_ (−815 mV), suggesting a potential for partial re-passivation that was not observed in the other Al-alloys. At the same time, after 30 days, the LH-welded Al-alloy had the smallest (~114 mV) and the MIG-welded Al-alloy had the largest (~195 mV) difference between E_pit_ and E_rep_. These results suggest that the MIG-welded Al-alloy has a higher tendency to pitting corrosion than the LH-welded Al-alloy under the tested conditions.

Further insights into the stability of the surface oxide layer can be derived from another parameter: the active–passive transition potential, which is often visible as a distinctive “anodic nose” in the polarisation curve. This potential marks the point on the reverse scan where the current density undergoes a sharp decline. In the tested BM and MIG-welded Al-alloys, E_corr_ was more noble (positive) than this transition potential, which suggests that the alloy surface would remain in a stable passive state at its corrosion potential [38]. The exception was the LH Al-alloy after 7 days, where the relative positions of these potentials were consistent with its unique re-passivation behaviour.

The result obtained by reversed scan polarisation highlights a fundamental distinction in the re-passivation kinetics between the two welded Al-alloys. The LH-welded Al-alloy exhibited a sharp, abrupt drop in current density upon approaching the re-passivation potential (Figure 9b,e,h). This rapid decline signifies the ability to reform a protective passive layer quickly once the potential falls below the critical threshold for pit growth.

Conversely, the MIG-welded Al-alloy demonstrated a different behaviour. Its current density decreased gradually over a broad potential range before finally reaching E_rep_ (Figure 9c,f,i). This sluggish decay indicates that the reformation of its protective oxide film is a slower, less efficient process. Consequently, the passive layer that forms on the MIG-welded Al-alloy is inferred to be less stable and more sensitive to pitting corrosion compared to the layer on the LH-welded Al-alloy.

**Figure 10:**
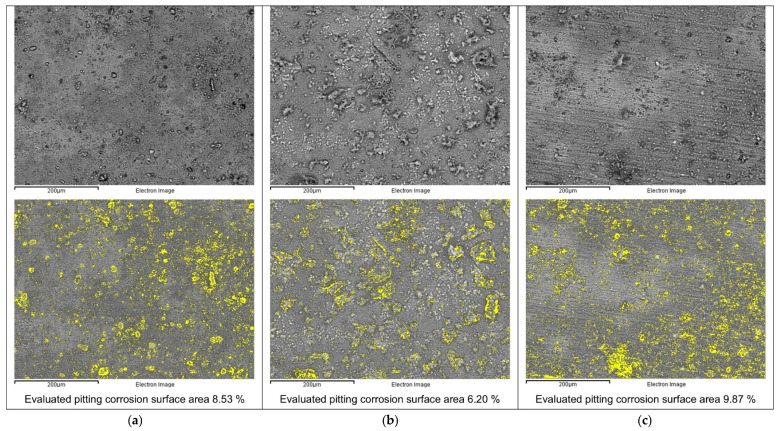
Surface morphology of the tested Al-alloy surface after potentiodynamic polarisation with a reverse scan (after an immersion time of 30 days in 3.5 wt.% NaCl); evaluation of pitting corrosion surface area (highlighted in yellow) with ImageJ processing on the selected sample locations: (**a**) BM, (**b**) LH-welded, and (**c**) MIG-welded specimens.

In order to check the possibility of pitting corrosion formation, the Al-alloy samples after potentiodynamic polarisation with reverse scans (30 days of immersion in the 3.5 wt.% NaCl solution) were analysed by SEM. Figure 10 shows the Al-alloy surface morphology after potentiodynamic polarisation with reverse scans (after 30 days of immersion in the 3.5 wt.% NaCl solution).

Based on the SEM micrographs in Figure 10, the surface morphology of the Al-alloys after potentiodynamic polarisation with reverse scans (after 30 days of immersion in the 3.5 wt.% NaCl solution) shows noticeable differences in corrosion damage.

The base Al-alloy surface (Figure 10a) exhibits a sparse distribution of corrosion products alongside numerous small pits and several larger ones, indicating active pitting corrosion. The evaluated pitting corrosion surface area on the selected location on the sample was 8.53%.

The MIG-welded Al-alloy presents a similar behaviour to the BM. After polarisation, its surface (Figure 10c) also appears largely free of precipitated products, and it is characterised by numerous deep pits, with the difference that these pits are not scattered randomly, but are clustered together, suggesting a higher pitting attack than in the case of the BM. The evaluated pitting corrosion surface area on the selected location on the sample was 9.87%.

In contrast, the surface of the LH-welded Al-alloy (Figure 10b) after polarisation is covered with a more substantial layer of corrosion products and shows significantly fewer pits. This behaviour indicates that, during the reverse scan, partial re-passivation occurred on the LH-welded Al-alloy surface, which is why its tendency to pitting corrosion was the lowest under the given conditions. The evaluated pitting corrosion surface area on the selected location on the sample was 6.20%.

#### 3.2.3. Electrochemical Impedance Spectroscopy Measurements

**Figure 11:**
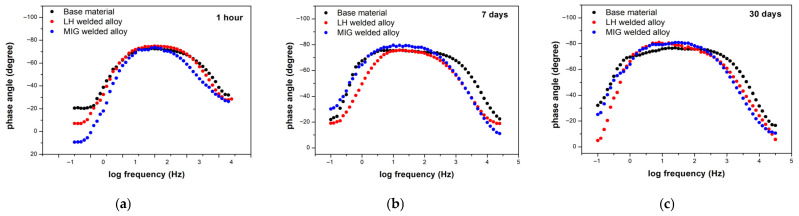
Bode phase diagram for the tested Al-alloys following immersion in a 3.5 wt.% NaCl solution: (**a**) 1 h, (**b**) 7 days, and (**c**) 30 days.

**Figure 12:**
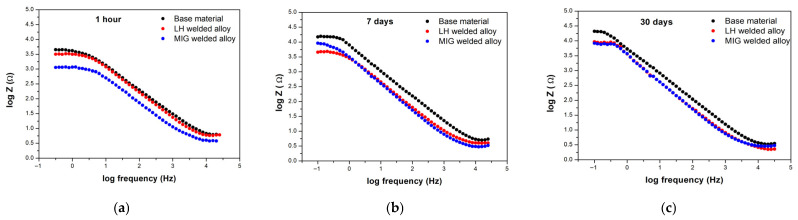
Bode magnitude plots for the tested Al-alloys following immersion in a 3.5 wt.% NaCl solution: (**a**) 1 h, (**b**) 7 days, and (**c**) 30 days.

**Figure 13:**
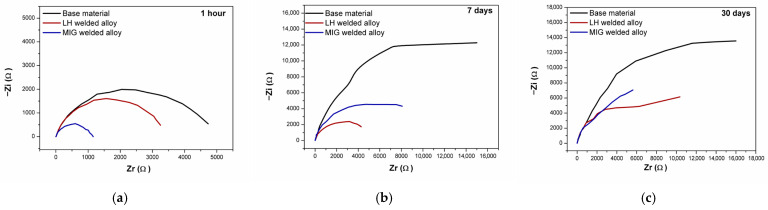
Nyquist plots for the tested alloys following immersion in a 3.5 wt.% NaCl solution: (**a**) 1 h, (**b**) 7 days, and (**c**) 30 days.

Electrochemical impedance spectroscopy (EIS) was performed at open circuit potential to investigate the interfacial behaviour and surface layer evolution of the Al-alloys over immersion periods of 1 h, 7 days and 30 days in a 3.5 wt.% NaCl solution. The results are presented as Bode phase (Figure 11a–c), Bode magnitude plots (Figure 12a–c), and Nyquist plots (Figure 13a–c).

The initial impedance measurement (Figure 11a) after one hour revealed a distinctive plateau in the Bode phase plot, with a maximum phase angle of approximately −75° observed in the intermediate frequency region near 100 Hz. This profile is a classic signature of capacitive behaviour, confirming the presence of a stable, protective film on the Al-alloy surface [52,53,54]. The high magnitude of this phase angle indicates further good corrosion resistance for all the tested Al-alloys under the specified conditions [52,55].

After 7 days of immersion (Figure 11b), the electrochemical response evolved to display two distinct time constants. A new time constant emerged in the lower intermediate frequency range (~10 Hz), maintaining a phase angle near −80°. Concurrently, a second time constant remained evident in the medium intermediate frequency region (~100 Hz), though with a maximum phase angle of approximately −75°. The persistent high phase angles (~70° to −80°) at lower frequencies continue to signify strong capacitive behaviour, and, thus, good protective properties. The appearance of the second time constant is attributed to the formation of an outer layer of corrosion products or a duplex oxide-based structure [52,56,57].

After 30 days (Figure 11c), both time constants shifted further towards lower frequencies (~6–7 Hz and 70–100 Hz, respectively), though the phase angle values remained largely unchanged. This suggests stabilisation of the surface layers without significant loss of the protective character.

The Bode magnitude plots (Figure 12a) revealed that, at 1 h, the low frequency impedance modulus (log Z) for all the Al-alloys fell within 3.0–3.5 Ω·cm^2^, with the MIG-welded Al-alloy exhibiting the lowest value. After 7 and 30 days (Figure 12b,c), the impedance increased slightly, reaching log Z values between 3.6 and 4.3 Ω·cm^2^, indicating improved corrosion resistance over time. The base Al-alloy showed the highest impedance consistently, while both welded Al-alloys converged to similar, slightly lower values after 30 days.

The slope of the magnitude plots in the intermediate frequency region was approximately −0.8 for all samples, suggesting that the surface layer behaves as a non-ideal capacitor with some ionic permeability, likely due to the porous nature of the oxide/corrosion product layer [58,59].

Across all the immersion periods, the high frequency region (1–100 kHz) showed consistent behaviour. The Bode magnitude plots exhibited a stable plateau, while the phase angle approached 0° (Figure 12a–c). This response is a direct fingerprint of the solution (electrolyte) resistance and showed no significant variation between the measurements.

The shape of the Nyquist plots evolved significantly with increasing immersion time, as illustrated in Figure 13a–c. A detailed analysis of this evolution, and the underlying electrochemical processes it represents, can be achieved by fitting an appropriate equivalent circuit model to the impedance data for each alloy under the given conditions.

The analysis of the impedance spectra revealed a non-ideal, frequency-dependent response (Figure 13a–c). To represent this behaviour accurately, a constant phase element (CPE, denoted as Q) was incorporated into the circuit model. The corresponding double-layer capacitance (C) was subsequently calculated from the CPE parameters using the standard conversion equation [54,58,59].

**Figure 14:**
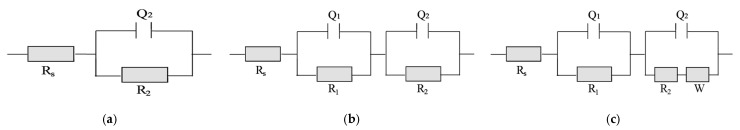
Equivalent circuit for the tested alloys following immersion in a 3.5 wt.% NaCl solution: (**a**) 1 h, (**b**) 7 days, and (**c**) 30 days.

To interpret the EIS data quantitatively, different equivalent circuits were used to model the electrochemical interface. After 1 h, a simple Randles circuit (Figure 14a) provided a good fit, where R_s_ represents the solution resistance, R the charge transfer resistance, and Q a constant phase element. After 7 days, a two-time constant model was required (Figure 14b), reflecting the development of a dual-layer structure. In this refined model, the elements R_1_ and Q_1_ (converted to C_1_) characterise the outer, porous corrosion product layer. The components R_2_ and Q_2_ (C_2_) describe the properties of the inner, barrier oxide layer [54]. After 30 days, the best fit was achieved by incorporating a Warburg element (W) into the circuit, indicating the onset of diffusion-controlled processes (Figure 14c).

The need to apply different models during modelling indicates that the properties of the layer on the surface of the Al-alloy changed with the increasing immersion time in the 3.5 wt.% NaCl solution.

Table 8 summarises the key parameters extracted from the equivalent circuit models used to fit the EIS data. These values correspond to the electrochemical behaviour of the Al-alloy after immersion periods of 1 h, 7 days, and 30 days in a 3.5 wt.% NaCl solution. The fitting quality was evaluated by chi-squared value, which, in these calculations, ranged from 0.0001 ± 0.004 to 0.0001 ± 0.03.

Upon the initial exposure to the 3.5 wt.% NaCl solution, the electrochemical interface is described accurately by a simple one-time constant model (Table 8). At this stage, the BM demonstrated the most favourable corrosion properties, registering the highest polarisation resistance (R = 5.0 kΩ cm^2^), and the MIG-welded Al-alloy exhibited the lowest resistance (R = 1.1 kΩ cm^2^). This ranking is supported by the double-layer capacitance (C) values, which were lowest for the base Al-alloy and highest for the LH-welded Al-alloy.

Following 7 days of immersion, the impedance data required a more complex model incorporating a second time constant. All the Al-alloys showed a substantial increase in overall resistance compared to the 1 h measurement, while the capacitance of the inner barrier layer (C_2_) increased in the case of BM and decreased in the case of welded Al-alloys (much more significantly in the case of the LH-welded Al-alloy). This trend suggests that the surface becomes less active, indicating that welded Al-alloys require a longer duration to establish an effective protective oxide layer compared to the BM.

The performance hierarchy established initially did not persist, with the base Al-alloy maintaining the highest corrosion resistance, but the LH-welded Al-alloy now has the lowest.

A consistent finding across all the samples was the superior quality of the inner oxide layer compared to the outer layer. This was evidenced by higher resistance (R_2_ > R_1_) and significantly lower capacitance (C_2_ < C_1_) values. The good properties of this inner barrier layer are primarily responsible for the Al-alloy’s corrosion protection during this period [54].

The electrical properties of the outer layer, specifically its high capacitance (C_1_) and low resistance (R_1_), are indicative of a hydrated, porous structure permeated by electrolyte ions. The ingress and concentration of aggressive ions, particularly chloride, within this outer layer, can destabilise the underlying barrier oxide, creating conditions favourable for the initiation of pitting corrosion.

After 30 days of immersion, the impedance spectra for all the Al-alloys exhibited a low-frequency diffusion tail, necessitating the inclusion of a Warburg element (W) in the equivalent circuit model to account for the mass transport limitations. The calculated Warburg impedance values were consistently small and similar across all the tested materials.

Compared to the 7-day data, the resistance of the inner barrier layer (R_2_) showed a slight decrease for the BM and MIG-welded Al-alloys. In contrast, in the case of the LH-welded Al-alloy, an increase in R_2_ was registered, which suggests that, in the case of the LH-welded Al-alloy, the internal oxide layer retains and even improves its protective properties after 30 days, while the same cannot be said for the BM and MIG-welded Al-alloys.

Consequently, after 30 days, the two welded Al-alloys demonstrated comparable resistance values for both the outer and inner layers. A key distinction, however, is that the LH-welded Al-alloy exhibited lower capacitance values for both layers, with the difference being particularly pronounced for the inner layer capacitance (C_2_).

The notably higher inner layer capacitance for the MIG-welded Al-alloy (26.15 mF/cm^2^ vs. 1.38 mF/cm^2^ for LH-welded) is a critical finding. This increase in C_2_ (not just C_1_) suggests that, in the MIG-welded sample, electrolyte ions, particularly chlorides, have penetrated the outer porous layer and begun to incorporate into the inner barrier oxide. This incorporation degrades the protective quality of the inner layer, reducing its resistance to uniform corrosion and increasing its susceptibility to localised pitting attack substantially.

## 4. Conclusions

The electrochemical behaviour of BM, LH-welded, and MIG-welded Al-alloys was investigated in a 3.5 wt.% NaCl solution over immersion periods of 1 h, 7 days, and 30 days using electrochemical techniques and surface analysis. The following conclusions can be drawn:

The potentiodynamic polarisation measurements indicate that the welding process decreased the initial corrosion resistance of the Al-alloys, with the MIG-welded Al-alloy exhibiting the poorest performance, which resulted in the highest initial corrosion current density and the lowest polarisation resistance.

- The corrosion resistance against uniform corrosion for all the Al-alloys improved with the immersion time, as evidenced by a significant decrease in the corrosion current density and an increase in the polarisation resistance after 7 and 30 days. This improvement is attributed to the formation and stabilisation of surface oxide/corrosion product layers.
- Polarisation with the reverse scan registered that the layer formed on the surface after 1 h, 7-day, and 30-day exposure periods in a 3.5 wt.% NaCl solution could not protect the tested Al-alloys completely against pitting corrosion. The protective properties of the formed layer for all the Al-alloys decreased as a function of time.
- The MIG-welded Al-alloy reforms the passive layer more slowly over a wider range of potentials before reaching the re-passivation potential than the LH-welded Al-alloy, and therefore, the passive layer that forms on the MIG-welded Al-alloy is less stable and more sensitive to pitting corrosion
- The surface morphology analysis after potentiodynamic polarisation, including reverse scans (after 30 days of immersion in the 3.5 wt.%NaCl solution), confirmed the electrochemical results. The surface of the BM and MIG-welded Al-alloy indicated active pitting corrosion, while partial re-passivation occurred in the LH-welded Al-alloy during the reverse scanning.
- The EIS analysis indicated the formation of a duplex surface layer over time, characterised by two time constants after 7 and 30 days. The MIG-welded Al-alloy exhibited the highest inner layer capacitance after 30 days, suggesting significant ion penetration and greater susceptibility to pitting corrosion.

Our results show the hierarchy BM > LH > MIG for resistance to both uniform and localised corrosion consistently [38]. The rationale is straightforward: LH welding tends to reduce porosity and microstructural heterogeneity versus arc welding, which lowers ionic ingress and stabilises the barrier layer during long-term exposure [35]. We added an EIS-based, quantitative picture—after 30 days, the MIG showed systematically higher C_2_ and lower R_2_—which we link directly to the increased pitting susceptibility [41]. This provides a mechanism-oriented, numerically supported framework to guide the process selection and surface post-treatment for the EN AW-5454-D Al-alloy.

## Figures and Tables

**Table 1:** The nominal chemical composition of the EN AW-5454-D Al-alloy (in wt.%), as specified by the manufacturer [42].

Thickness [mm]	Element	Si	Fe	Cu	Mn	Mg	Cr	Zn	Ti	Al
4.0		0.18	0.32	0.05	0.76	2.85	0.05	0.04	0.02	95.73

**Table 2:** The chemical composition of the Al5554 welding wire (in wt.%) [42].

Cr	Cu	Fe	Mg	Mn	Si	Ti	Zn	Al
0.079	0.0019	0.148	2.83	0.609	0.057	0.088	0.0018	Rest

**Table 3:** Technological parameters for LH welding and MIG welding.

Welding Parameter	Value	Value
Welding process	LH	MIG-PMC
Welding position	PA—Flat position	PA—Flat position
Gap Laser–MIG	2 mm	-
Sequence	Laser Master, MIG Slave	-
Stickout	15 mm	15 mm
Fibre diameter	0.6 mm	-
Spot size	0.9 mm (no defocus)	-
Collimation length	200 mm	-
Focal length	300 mm.	-
Optic	5° in pulling position	-
Shielding gas	100% Ar 5.0	100% Ar 5.0
Gas flow	15 L/min	15 L/min
Welding speed	300 cm/min	96 cm/min
Wire feed speed	10.5 m/min	5.5 m/min
Welding current	147 A	181 A
Voltage	18.1 V	20 V
Laser power	5000 W	-
Type of welding current and polarity	DC—Pulse	DC—Pulse
AC balance	+2	0
Arc length	−0.2	0

**Table 4:** EDX analysis results of the LH weld cross-section (in wt.%).

Spectrum	C	O	Mg	Al	Cr	Fe	Mo	Total
Spectrum 1	13.29	12.69	1.64	72.38	-	-	-	100
Spectrum 2	-	11.62	1.98	86.40	-	-	-	100
Spectrum 3	-	-	1.22	77.37	-	21.41	-	100
Spectrum 4	-	7.72	1.53	62.72	1.73	20.05	6.25	100
Spectrum 5	-	14.28	-	53.97	4.63	22.67	4.45	100
Spectrum 6	-	13.59	-	44.10	2.19	19.75	20.38	100
Mean	2.22	9.98	1.06	66.16	1.43	13.98	5.18	100
Std. Dev.	5.43	5.41	0.86	15.63	1.85	10.88	7.91	
Max.	13.29	14.28	1.98	86.40	4.63	22.67	20.38	
Min.	0	0	0	44.10	0	0	0	

**Table 5:** Air plasma technological parameters.

Parameter	Value	Unit
Supply air pressure	3.6	bar
Voltage	300	V
Electric current	11.7	A
Frequency	21	kHz
Plasma cycle time	100	%
Nozzle distance from the surface	4	mm
Nozzle/transition speed	25	mm/s
Nr. of transitions	4	-

**Table 6:** EDX analysis results of the BM samples (in wt.%).

Spectrum	O	Mg	Al	Si	Mn	Total
Spectrum 1	3.28	2.93	92.83	-	0.96	100
Spectrum 2	2.56	2.96	93.53	-	0.95	100
Spectrum 3	2.24	2.73	94.02	-	1.01	100
Spectrum 4	4.85	3.63	90.13	1.39	-	100
Spectrum 5	4.93	3.82	90.44	-	0.81	100
Spectrum 6	6.08	2.07	91.04	-	0.81	100
Mean	3.99	3.02	92.00	0.23	0.76	100
Std. Dev.	1.52	0.63	1.67	0	0.38	
Max.	6.08	3.82	94.02	1.39	1.01	
Min.	2.24	2.07	90.13	0	0	

**Table 7:** Parameters of the polarisation measurements of the tested Al-alloys for different immersion times in a 3.5 wt.% NaCl solution.

Al-Alloy	1 h			7 Days			30 Days		
	E_corr_ (mV)	j_corr_ (µA/cm^2^)	R_p_ (kΩ/cm^2^)	E_corr_ (mV)	j_corr_ (µA/cm^2^)	R_p_ (kΩ/cm^2^)	E_corr_ (mV)	j_corr_ (µA/cm^2^)	R_p_ (kΩ/cm^2^)
BM	−750	3.80	4.55	−795	0.70	21.43	−794	0.78	22.0
LH-welded	−743	4.63	3.05	−853	1.05	11.98	−815	1.16	14.7
MIG-welded	−806	18.15	1.60	−761	0.88	18.10	−816	1.45	7.70

The average value of the obtained parameters is within the limits of variability of 0.10–0.25%.

**Table 8:** EIS parameters of the Al-alloy at different immersion times in a 3.5 wt.% NaCl solution.

Al-Alloy	R_s_ (Ωcm^2^)	R_1_ (kΩcm^2^)	C_1_ (mF/cm^2^)	n_1_	R_2_ (kΩcm^2^)	C_2_ (mF/cm^2^)	n_2_	W (kΩcm^2^)
			1 h					
BM	5	---	---	---	5.0	10.98	0.85	---
LH-welded	5	---	---	---	3.4	24.28	0.95	---
MIG-welded	3	---	---	---	1.1	16.69	0.95	---
			7 days					
BM	3	10.0	670.8	0.85	31.0	17.71	0.90	---
LH-welded	3	4.0	556.0	0.86	4.9	14.43	0.93	---
MIG-welded	3	7.5	341.7	0.91	9.0	13.58	0.94	---
			30 days					
BM	5	5.0	197.2	0.92	25.0	5.07	0.93	0.35
LH-welded	5	6.0	273.1	0.92	9.0	1.38	0.93	0.40
MIG-welded	5	5.0	335.0	0.93	8.0	26.15	0.94	0.30

The average value of the obtained parameters is within the limits of variability of 0.10–0.25%.

## Data Availability

The original contributions presented in this study are included in the article. Further inquiries can be directed to the corresponding author.

## Abbreviations

The following abbreviations are used in this manuscript:

BM	Base material
BSE	Backscattered electron
CW	Continuous welding
E_corr_	Corrosion potential
EDX	Energy-dispersive X-ray
EIS	Electrochemical impedance spectroscopy
FSW	Friction stir welding
HAZ	Heat-affected area
j_corr_	Corrosion current density
LH	Laser hybrid welding
MIG	Metal inert gas
OCP	Open circuit potential
Rₚ	Polarisation resistance
SCE	Saturated calomel electrode
SE	Secondary electron
SEM	Scanning electron microscope
